# Guillain-Barré syndrome as a cause of acute flaccid paralysis in Iraqi children: a result of 15 years of nation-wide study

**DOI:** 10.1186/1471-2377-13-195

**Published:** 2013-12-10

**Authors:** Jagar Jasem, Kawa Marof, Adnan Nawar, Yosra Khalaf, Sirwan Aswad, Faisal Hamdani, Monirul Islam, Andre Kalil

**Affiliations:** 1 School of Medicine/ Faculty of Medical Sciences/ University of Duhok, Nakhoshkhana Street, Duhok, Kurdistan Region, Iraq; 2 Internal Medicine, Ohio State University, Columbus, Ohio, USA; 3 Directorate of Preventive Health Affairs, Directorate General of Health, Mazi Street, Duhok, Kurdistan Region, Iraq; 4 National Communicable Disease Control, Ministry of Health, Bab Al Mudam Area, Baghdad, Iraq; 5 AFP Surveillance Laboratory, Ministry of Health, Bab Al Mudam Area, Baghdad, Iraq; 6 College of Public Health, University of Nebraska Medical Center, Omaha, Nebraska, USA; 7 Division of Infectious Diseases/Department of Internal Medicine, University of Nebraska Medical Center, Omaha, Nebraska, USA

## Abstract

**Background:**

Guillain-Barré syndrome (GBS) is the most common cause of acute flaccid paralysis (AFP) in the post-poliomyelitis eradication era. This is the first study done to identify the epidemiology, clinical features, and outcome of GBS in Iraqi children over 15 years.

**Methods:**

The surveillance database about AFP cases < 15 years reported during January 1997-December 2011 was used.

**Results:**

GBS represented 52.5% of AFP cases, with an incidence of 1.33 case/100,000 population < 15 years/year. There was a higher incidence in the Southern provinces, age group 1–4 years, males, and outside the capital city of province, with no significant seasonal variations (p = .22). Survival probability after the 1 year of onset for those with respiratory muscle involvement was .76 (95% CI: .60-.86), versus .97 (95% Cl: .96-.98) for those who did not develop it (*p* < .001); and .97 (95% CI: .96-.98) for those living inside the capital city, versus .94 (.93-.95) for those living outside (*p* = .001). Cumulative incidence of residual paralysis for patients living inside the capital city was .21 (95% CI: .18-.24), versus .27 (95% CI: .25-.29) for those living outside (*p* < .001).

**Conclusions:**

The incidence, age and gender distribution, and seasonality of GBS among Iraqi children is similar to those reported from other previous studies. It is the most important cause of AFP, especially in those between the age of 1 to 4 years living in rural areas.

## Background

Guillain-Barré syndrome (GBS) is mostly an acute inflammatory demyelinating ascending polyradiculoneuropathy [1-3]. Flu-like illness or gastroenteritis precedes the onset of paralysis by 6 weeks in about two-thirds of patients [3]. The culprit infectious agent often remains unrecognized, but *Compylobacter jejuni Mycoplasma pneumonia*, and cytomegalovirus are commonly reported triggering pathogens. Molecular mimicry between structural components of both pathogens and myelin sheath of peripheral nerves, with subsequent cross-reaction of antibodies with the latter, is a commonly proposed hypothesis for the pathogenesis of disease [3-6].

Worldwide, GBS is considered the most common cause of acute flaccid paralysis in the post-poliomyelitis eradication era [5-14]. It affects people in various geographical locations and virtually all age groups [15]. Similar studies about the epidemiology and clinical features of GBS yield a wide range of minor to major differences [4,10,15-18]. Information about the epidemiology of the disease can give insights into the changing patterns of the disease incidence following exposure to new potential provoking factors [16]. Measuring the outcome of the disease in terms of mortality and morbidity can help direct attention towards modifying the risk factors of adverse disease outcomes. This is the first study done to identify the epidemiology, clinical features, and outcome of GBS in Iraqi children over 15 years of study.

## Methods

The surveillance database about acute flaccid paralysis (AFP) cases under the age of 15 reported from Iraq during January 1997 to December 2011 was used in the current study. AFP surveillance is an essential strategy of the Polio Eradication Initiative adopted by the World Health Organization (WHO) in 1988. AFP is defined as “any child under 15 years of age with acute flaccid paralysis (including GBS) or any person of any age with paralytic illness if polio is suspected”. AFP information is routinely included in the weekly and monthly reporting system from the Preventive Health Department (PHD) in the Directorate General of Health (DGoH) of every Iraqi province, even if there is no reported case (routine surveillance “zero-reporting”). Notifications are done via mailing the standard communicable notification forms designed by the Iraqi Ministry of Health to the Communicable Disease Control Unit (CDCU) of the regional PHD. Active surveillance for the suspected cases of AFP is ideally done within 48 hours via a designed investigating team from the CDCU visiting the reporting sources (hospitals, rehabilitation centers, or private clinics). Case investigation is done using a WHO-standardized form [19].

Two stool specimens are collected from each suspected case with an interval of 24–48 hours between collections, given that no more than two months have elapsed since the onset of paralysis. Following collection, the specimens are kept in a cold box to be sent for the National Polio Laboratory in Baghdad. At least 60 days after the onset of paralysis, all surviving patients are re-examined by an expert committee for residual paralysis. The diagnosis of GBS was made based on clinical evaluation and cytochemical analysis of the cerebrospinal fluid retrieved via conducting a lumbar puncture test of the suspected cases.

There is no clear classification for “urban” and “rural” areas in Iraq. Hence, the most approximate encounter for that fact is via dividing each Province into areas inside and outside the Capital City of each; referring to more urban and more rural areas, respectively.

The study was approved by the Ethical Committee of the Faculty of Medical Sciences/University of Duhok, Duhok, Iraq and the Institutional Review Board (IRB) of the University of Nebraska Medical Center, Omaha, Nebraska, USA. The statistical analysis was done using SPSS 18 for Windows/MAC, (PASW® Statistics GradPack 18). Statistical analyses included: Chi-square test, univariate Kaplan-Meier survival analysis and stratified log-rank tests. All tests were two sided with .05 level of significance.

## Results

**Figure 1:**
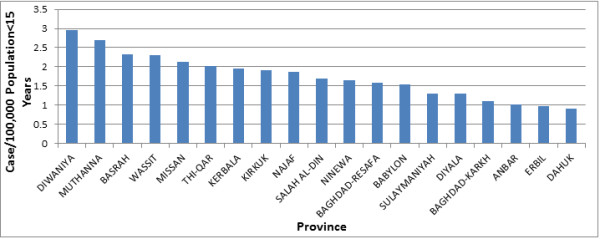
Incidence of Guillain-Barré syndrome per province, Iraq, 1997–2011.

**Figure 2:**
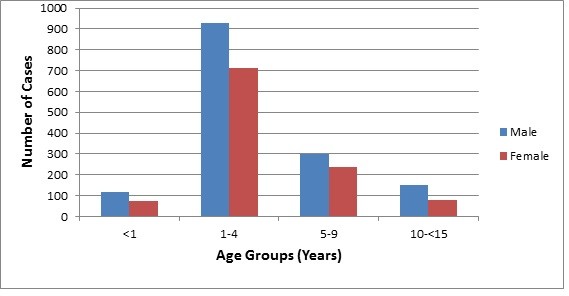
Number of cases with Guillain-Barré syndrome per age group, Iraq, 1997–2011.

**Figure 3:**
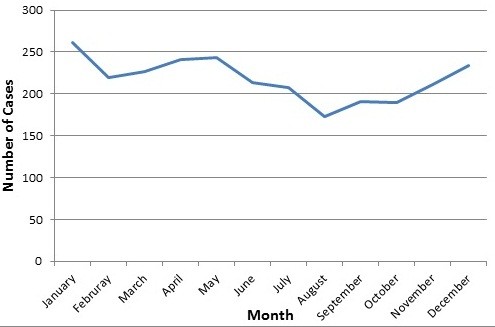
Number of cases with Guillain-Barré syndrome per month, Iraq, 1997–2011.

A total number of 5027 cases were reported from Iraq between January 1997 and December 2011. Out of these, 53 cases were excluded as they were not AFP (nutritional deficiencies and skeletal diseases). A total of 4974 cases of AFP were used in the final analysis. GBS represented more than half of the reported cases (N = 2611, 52.5%) (Table 1), corresponding to an annual incidence of 1.33 case/100,000 population under the age of 15 years (95% CI: .97-1.68). There was a higher incidence in the Southern provinces compared to the Central and Northern ones (Figure 1). The vast majority of cases belonged to the age group 1–4 years old (Figure 2). Male–female ratio was 1.35:1. About 65% of cases occurred outside the capital city of province of more rural social characteristics. Cases were reported throughout the year with the highest number being in January (261) and the lowest in August (173) (Figure 3). This monthly variation was not statistically significant (*p* = .22).

**Table 1:** Causes of acute flaccid paralysis in Iraqi children, 1997-2011

**Cause**	**Number (%)**
Guillain-Barré syndrome	2611 (52.5)
Traumatic neuritis	715 (14.4)
Meningitis/Encephalitis	292 (5.9)
Poliomyelitis	166 (3.3)
Myopathy	89 (1.8)
Hypokalemia	74 (1.5)
Unknown	568 (11.4)
Others	459 (9.4)
Total	4974 (100)

Fever was observed in 1358 (55.4%) of cases, and progression of paralysis to the maximum within 4 days of onset occurred in 2450 (97.1%) of cases. At least 60 days from the onset of paralysis, 619 (24.6%) of cases had residual paralysis and 118 (4.8%) others died (Table 2).

**Table 2:** Selected clinical characteristics of cases with Guillain-Barré syndrome, Iraq, 1997-2011

**Features**	**Cases (%)**
**Fever at the onset of paralysis**	2449 (93.8)
Yes	1358 (55.4)
No	1091 (44.5)
**Progression to the maximum paralysis within 4 days of onset**	2523 (96.6)
Yes	2450 (97.1)
No	73 (2.9)
**Residual paralysis at least 60 days from onset**	2343 (89.7)
Yes	619 (26.4)
No	1724 (73.6)
**Survival at least 60 days from onset**	2461 (94.2)
Alive	2343 (95.2)
Dead	118 (4.8)

**Figure 4:**
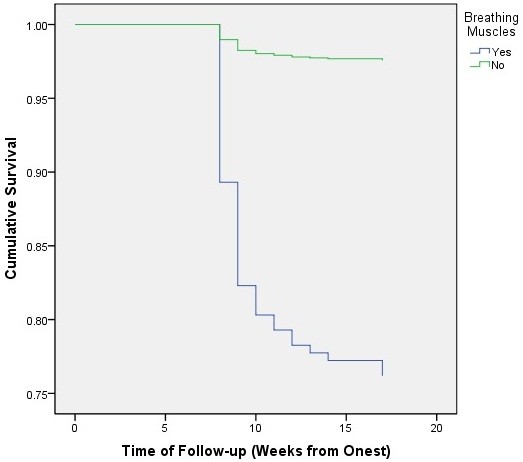
Cumulative survival of cases with Guillain-Barré syndrome based on history of respiratory muscle paralysis, Iraq, 1997–2011.

**Figure 5:**
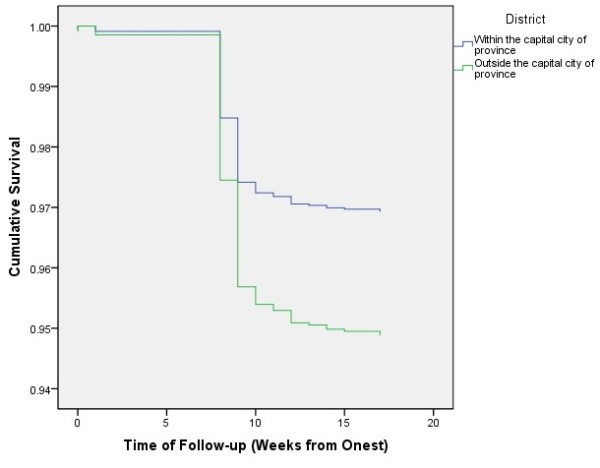
Cumulative survival of cases with Guillain-Barré syndrome based on their household location within the province, Iraq, 1997–2011.

**Figure 6:**
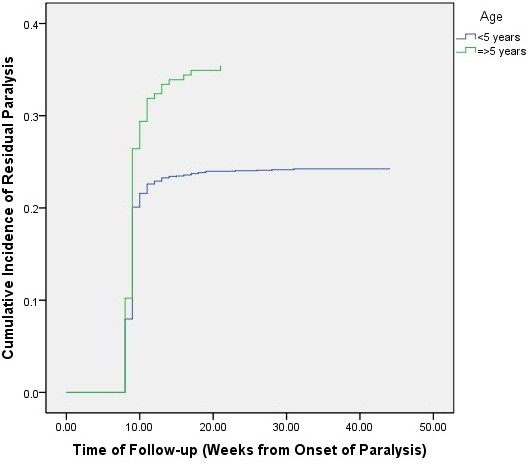
Cumulative incidence of residual paralysis among cases with Guillain-Barré syndrome, Iraq, 1997–2011.

**Figure 7:**
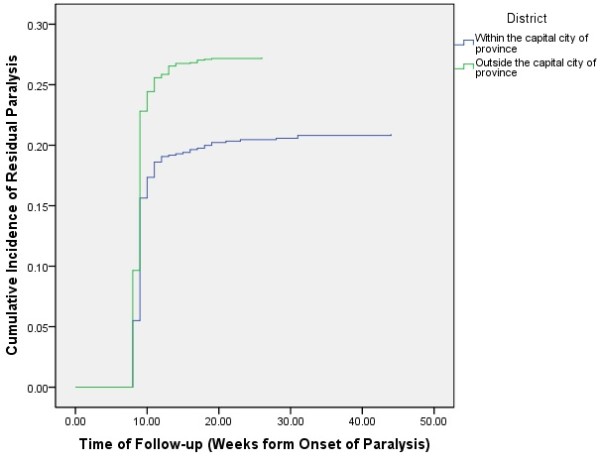
Cumulative incidence of residual weakness among cases with Guillain-Barré syndrome based on their household location within the province, Iraq, 1997–2011.

The probability of survival after the 1 year of onset for those with respiratory muscle involvement was .76 (95% CI: .60-.86), versus .97 (95% Cl: .96-.98) for those who did not develop respiratory muscle weakness (*p* < .001) (Figure 4). Kaplan-Meier analysis of household location, gender, and age showed that only household location was significantly associated with the decreased probability of survival. The cumulative survival for patients living inside the capital city of province was .97 (95% CI: .96-.98), versus .94 (.93-.95) for those living outside the capital city of province (*p* = .001) (Figure 5). This effect was eliminated when stratified log-rank test was performed for both respiratory muscle paralysis and household location (*p* = .21). Likewise, Kaplan-Meier analysis of household location, gender, and age showed that both household location and age were significantly associated with increased cumulative incidence of residual paralysis. The cumulative incidence of residual weakness increased with age was: .22 (95% Cl: -.20-.24) for those below the age of 5 years and .35 (.29-.41) for those above the age of 5 years (*p* = .001) (Figure 6). The cumulative incidence of residual paralysis for patients living inside the capital city of province was .21 (95% CI: .18-.24), versus .27 (95% CI: .25-.29) for those living outside the capital city of province (*p* < .001) (Figure 7). This effect was maintained when stratified log-rank test was performed for both age and household location (*p* = .001).

## Discussion

The annual incidence among Iraqi children was 1.33/100,000 population under the age of 15 years. This rate is at the upper limit of the reported international range of .34-1.34 cases/100,000/year among children aged <15 years [16]. Figures as high as 5 cases/100,000/year have been reported from some districts in Bangladesh [7].

Unlike other autoimmune diseases, males are typically affected more than females [10,16,20,21]. However, male predilection is found in other immune-mediated peripheral neuropathies like chronic inflammatory demyelinating polyneuropathy, multifocal motor neuropathy and Miller-Fisher syndrome [4]. Our study showed a male–female ratio of 1.3:1. Individual studies report ratios of 1.5 to 2.7 males to 1 female [20]. However, estimated 662 children reported in different studies have shown a male–female ratio of 1.3:1. Likewise, a total of 1607 patients of all ages reported in different studies have shown a similar ratio of 1.3:1 [20]. Pooled data about the cases of GBS in Latin America showed the exact same ratio [21]. This indicates that the higher preponderance of males in individual studies is likely to be the product of the confounding effect of sample size (Simpson’s paradox) [20].

In accordance with other reports, children 1–4 years old were the most commonly affected age group with GBS in our study [9,10,22,23]. This is believed to be due to their relatively high susceptibility to infections in this age group and the increased susceptibility to the young myelinated peripheral nerves to demyelination [22,23]. Southern provinces have a statistically significant higher incidence of GBS compared to both Central and Northern ones. Although no specific reason was found, geographic variation in the incidence of disease in Iraq may be due to differences in the infection rates and climate among the different regions [7,9].

Many studies have addressed the seasonality in the incidence of GBS, [9,15,16,24,25] only a few demonstrated a significant seasonal trend [18,24,26,27]. The lack of clear seasonal association may be due to the fact that the respiratory and enteric infections that precede GBS have opposite seasonal patterns [17]. The higher number of cases reported in winter and spring in our study is similar to reports from Southern Iran and Kuwait [18,28]. In general, different countries have different clustering patterns of cases, which might reflect the heterogeneity of the infectious agents that trigger the disease. The same reason may explain the higher number of cases reported outside the capital city of provinces, dominated by rural areas.

Although not classically attributed to the disease process of GBS [9,29], fever at the onset of paralysis is reported from previous studies and may be attributed to the effect of the triggering infectious disease [9,21,30]. Our study revealed that 55.4% of cases had fever at the onset of paralysis. As it might be expected from a study that is primarily concerned about AFP surveillance as a sensitive measure to detect cases of poliomyelitis, specific details about the progression pattern of weakness, sensory involvement, and dysautonomia in patients with GBS were not recorded in the study.

Although information about respiratory muscle involvement were only recorded for 1166 (44.7%) of patients, cumulative survival was significantly lower in those with respiratory muscle involvement. Likewise, the cumulative survival was significantly lower among those living outside the capital city of province. However, the latter association was insignificant when stratified by respiratory muscle involvement. This indicates that household location is not an independent cause of death per se, but rather causes death via respiratory paralysis, which plays a “mediating” effect. Furthermore, the association between household location and respiratory paralysis was insignificant too (*p* = .12), indicating that although respiratory paralysis occurs equally among those living inside and outside the capital city of province; the latter group are more likely die from it. This finding may be due to one or more of: differences of the inciting factors, delayed diagnosis in the rural areas, and inaccessibility to the required intensive care in those areas. Respiratory intensive care units do not exist in the vast majority of hospitals serving the rural communities in Iraq.

The risk of residual paralysis was higher among children above the age of 5 years. In general, the prognosis of GBS is shown to be better among younger age groups, although comparisons were generally made with adults [31-33]. In the largest prospective study by Korinthenberg et al., 96% of children were either asymptomatic or had minor symptoms at the end of the 288-day observation period [34]. This might reflect poorer axonal outgrowth and regeneration and less effective remyelinization process with increasing age [32]. Again, lower quality of and access to health care might underlie the increased risk of residual paralysis among cases residing outside the capital city of province.

The current study is the first to be done in Iraq involving analysis of nation-wide data over 15 years of study. It presents information about the overall incidence of disease, as well as the differences in disease incidence with time, age, gender, season, province of residence, and household location within the province of residence. It also provides information about the case-fatality rate of the disease and the likelihood of developing residual weakness following the resolution of acute disease, with emphasis on gender, age, and household location differences in those adverse outcomes. The sensitivity of AFP investigation was 2.5 cases/100,000 population below the age of 15 (above the WHO-recommended cut-off level of 1/100,000) and completeness of reporting was 96% (above the above the WHO-recommended cut-off level of 80%). These two performance indicators imply that the vast majority of GBS cases among children below15 years were effectively detected and recorded in the current database. However, since the data were collected for the purpose of acute flaccid surveillance as a strategy for detecting cases of poliomyelitis, the study provides limited information about the clinical features and subtypes of disease. Future studies are needed to at least identify the main 4 main subtypes of GBS, including: acute inflammatory demyelinating polyradiculoneuropathy (AIDP), acute motor axonal neuropathy (AMAN) and acute motor and sensory axonal neuropathy (AMSAN), and Miller Fisher syndrome [16]. Likewise, no data were available about the treatment modalities used for treating GBS cases. However, IVIG therapy remains largely unavailable in Iraq. Information about the history of preceding respiratory and gastrointestinal infection needs to be documented in future studies.

## Conclusions

The incidence, age and gender distribution, and seasonality of GBS among Iraqi children is similar to those reported from other previous studies. It is the most important cause of AFP, especially in those between the age of 1 to 4 years living in rural areas.

## Competing interests

The authors declare that they have no competing interests.

## Authors’ contributions

JJ has made substantial contributions to design of the study, data analysis, and writing and submitting the manuscript. KM, AN, YK, SA, and FH have contributed in data acquisition and manuscript revision. MI and AK have supervised study design and data analysis, as well as critically revised the manuscript. All authors have read and approved the final manuscript.

## Pre-publication history

The pre-publication history for this paper can be accessed here:

http://www.biomedcentral.com/1471-2377/13/195/prepub
